# Screening of coexpression genes of immune cells in breast cancer tissues

**DOI:** 10.1097/MD.0000000000036211

**Published:** 2024-01-05

**Authors:** Yuan-Yuan Zhang, Yi-Min Gan

**Affiliations:** a Department of Laboratory Medicine, The Affiliated Huaian No.1 People’s Hospital of Nanjing Medical University, Huai’an, Jiangsu, China; b Blood Research Laboratory, The Affiliated Huaian No.1 People’s Hospital of Nanjing Medical University, Huai’an, Jiangsu, China.

**Keywords:** breast cancer, immune cells, immune microenvironment, prognosis, public database

## Abstract

This study aimed to investigate immune cell infiltration (ICI) in breast cancer tissues and its impact on the prognosis of patients. The whole transcriptome sequencing data sets of breast tissue (GSE126125, GSE190275 and GSE45498) were downloaded from Gene Expression Omnibus database. Data sets, including 281 breast cancer tissue samples and 59 normal breast tissue samples. In this study, the CIBERSORT algorithm was used to calculate the infiltration content of 22 immune cells subtypes in breast cancer tissues and normal breast tissues. The ICI between normal and breast cancer tissue samples was examined through the Rank-sum test. Furthermore, Kaplan–Meier and the log-rank test were used for survival analysis. Univariate and multivariate COX analysis was used to screen the prognostic risk factors of breast cancer based on ICI. The correlation between 22 kinds of immune cells was analyzed by the Pearson test. The results of univariate COX analysis indicated that resting dendritic cells, eosinophils, resting mast cells, monocytes, and memory CD4 T cells resting were protective factors for the prognosis of breast cancer patients (hazard ratio [HR] < 1, *P* < .05). The activation of macrophage M0 and mast cells were also prognostic risk factors for breast cancer patients (HR > 1, *P* < .05). Besides, multivariate COX analysis showed that resting mast cells were independent protective factors for the prognosis of breast cancer patients (HR < 1, *P* < .05). Macrophage M0 and mast cell activation were independent risk factors for the prognosis of breast cancer patients (HR > 1, *P* < .05). High infiltration of macrophage M0 and activated mast cells is associated with poor prognosis. Meanwhile, macrophage M0 and activated mast cells promote breast cancer progression. Low infiltration of resting mast cells is associated with poor prognosis, which inhibits breast cancer progression.

## 1. Introduction

Breast cancer is one of the most common malignant tumors in women. More than half of them are at the advanced stage of diagnosis.^[1,2]^ Breast cancer has the highest mortality rate among women younger than 45 years old.^[3,4]^ Breast cancer is prone to distant metastasis, including transferring^[4,5]^ to the lung, liver, kidney and bone. Treating breast cancer is complex and expensive and requires long-term follow-up.^[6–9]^ At present, breast cancer treatment cannot achieve the desired effect. Therefore, patients need to face a high recurrence rate and drug resistance.^[10]^ With the development of medical technology, breast cancer treatment has gradually been systematized.^[11]^ Recently, immunotherapy has provided new ideas for treating breast cancer, especially for breast cancer in an advanced stage.^[12]^ For example, previous research suggested potential targets of immune checkpoint therapy, which included proteins such as cytotoxic T lymphocyte-associated antigen 4 and programmed cell death ligand 1 in breast cancer.^[13]^

The study of the breast cancer microenvironment indicates that the biological behavior of breast cancer cells is affected by the interaction between tumor cells and immune cells.^[14]^ There are 22 subtypes of immune cells, and different subtypes of immune cells have different effects on cancer cells. Inflammation and functional remodeling of immune cells in cancer tissues were one of the important characteristics of cancer, and immune cell remodeling in cancer tissues, in turn, affects the malignant evolution of cancer cells to a large extent. There were great differences in lymphocyte infiltration in different subtypes of breast cancer, among which Triple-negative breast cancer was accompanied by a large number of CD8 + T lymphocyte infiltration, which was closely related to good disease-free survival of patients. In contrast, regulatory T cell infiltration is predominant in ER + and HER2 + breast cancer tissues and is not associated with patient prognosis.^[14]^ Exploring the infiltration of immune cells in breast cancer can provide clues for explaining the mechanism of interaction between tumor and immune cells and finding new targets for immunological therapy. The purpose of this study was to explore the effects of different subtypes of immune cell infiltration (ICI) and different immune cell subtypes on prognosis in breast cancer.

## 2. Materials and methods

### 2.1. Data download

This study downloaded a complete set of transcriptome data sets (GSE126125, GSE190275, and GSE45498) of breast tissue from the Gene Expression Omnibus database. The data set consisted of 281 breast cancer tissue samples and 59 adjacent normal tissue samples. Batch correction and normalization were performed on the data set, and the RNA expression profile was summarized, logarithmically transformed, and combined into a matrix. For merging multiple data sets we first used the inSilicoMerging package, and then we used the ComBat_seq function in the SVA package for batch correction. At the same time, we downloaded the corresponding clinical data files of breast cancer tissue and extracted and collated clinical data, including age, gender, tumor stage, grading, survival time, and so on. The downloaded data is public and open. Therefore, approval from the local ethics committee is not required.

### 2.2. Calculation of ICI content

The CIBERSORT algorithm (https://cibersort.stanford.edu/) was used to calculate the infiltration of 22 immune cells in breast cancer tissue and normal breast tissue.^[15]^ 1000 permutations and combinations were run and calculated immune cells’ immersion content in tissue samples in R software. In this study, the CIBERSORT *P* value < .05 calculation results were retained.

### 2.3. Statistical analysis

The data used in this study were statistically analyzed by R software and related R packages. The infiltration of immune cells between normal breast tissue samples and breast cancer tissue samples was compared with the rank-sum test. Kaplan–Meier was used for survival analysis, and the log-rank method was used for the test. Based on the content of ICI, single factor and multivariate COX analysis were used to screen the prognostic risk factors of breast cancer. Pearson test was used to analyze the correlation between 22 immune cells. *P* < .05 means statistically significant.

## 3. Results

### 3.1. Difference of ICI between normal tissue and tumor tissue

**Figure 1.**
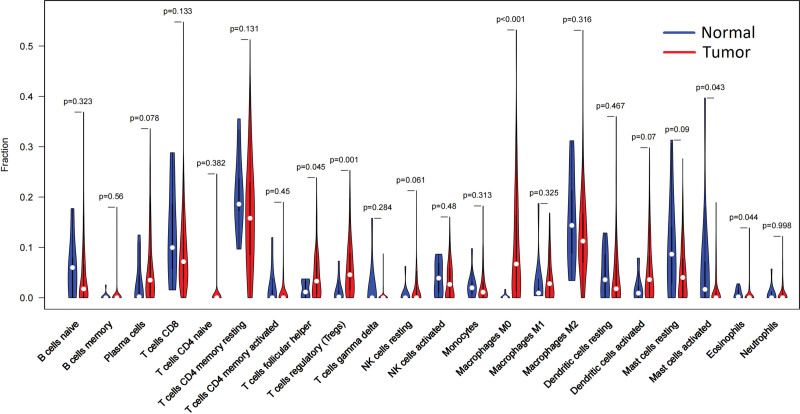
There was a difference in the infiltration of immune cells between normal breast tissue and breast cancer tissue. Blue represented normal tissue and red represented tumor tissue.

This study used the CIBERSORT algorithm to calculate the proportion of immune cells in normal breast tissue and breast cancer tissue samples. The infiltration of follicular helper T cells, regulatory T cells and macrophages M0 in breast cancer tissue was higher than that in normal breast tissues (*P* < .05). The mast cells and eosinophils in breast cancer tissues were less than those in normal tissues, and the difference was statistically significant (*P* < .05). As shown in Figure 1.

### 3.2. Correlation of immune cells in tumor samples

**Figure 2.**
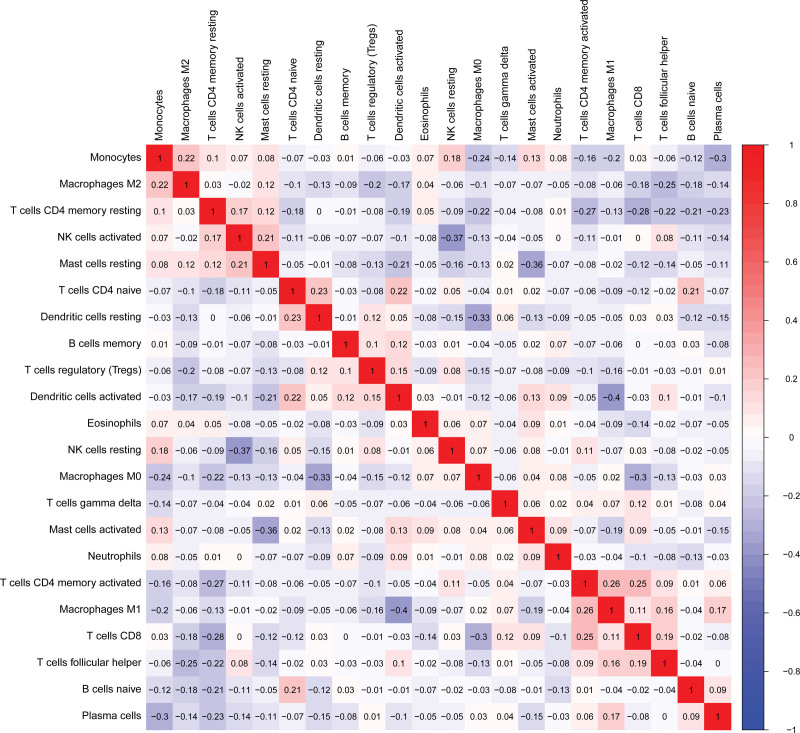
Correlation of immune cells in breast cancer samples. Red indicates positive correlation, and blue indicates negative correlation.

Correlation analysis illustrated a moderate or weak correlation between 22 kinds of immune cells. Macrophage M1 had the highest correlation with activated dendritic cells (r = −0.4, *P* < .05). As shown in Figure 2.

### 3.3. Immune cell content and survival analysis

**Figure 3.**
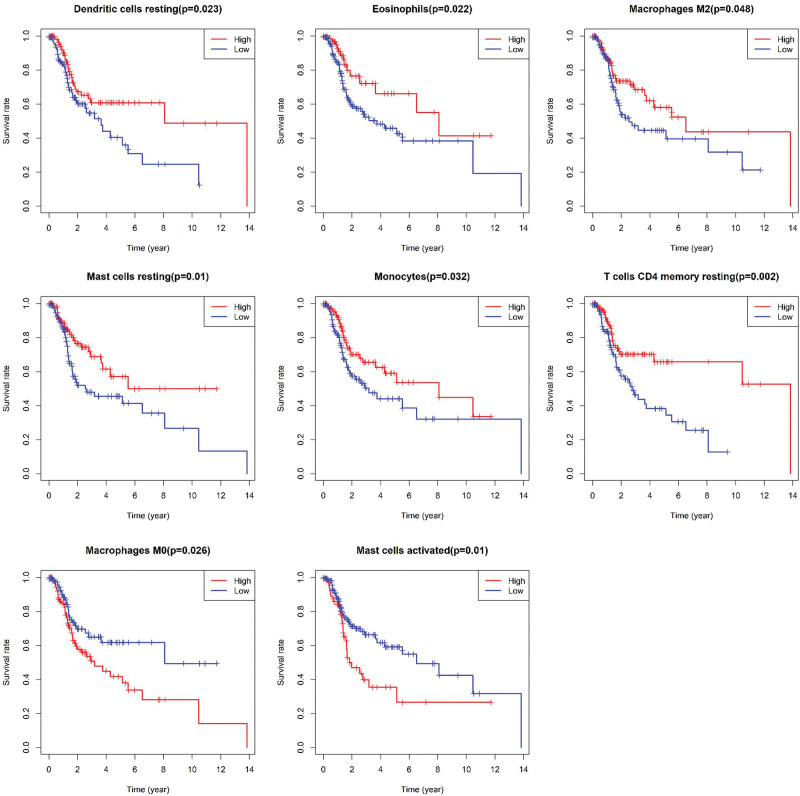
Survival analysis of immune cell infiltration.

Taking the median value of ICI content as the critical value, the samples were divided into high ICI groups higher than the median value and low ICI groups lower than the median value for the Kaplan–Meier survival curve in the log-rank test. The prognosis of the high infiltration group of resting dendritic cells was better than that of the low infiltration group (*P* = .023, Log-rank statistic = 3.868). The prognosis of the high eosinophil infiltration group was better than that of the low infiltration group (*P* = .022, Log-rank statistic = 3.002). The prognosis of the macrophage M2 high infiltration group was better than that of the low infiltration group (*P* = .048, Log-rank statistic = 2.534). The prognosis of the high infiltration group of dormant mast cells was better than that of the low infiltration group (*P* = .010, Log-rank statistic = 3.955). The prognosis of the high monocyte infiltration group was better than that of the low infiltration group (*P* = .032, Log-rank statistic = 2.576). The prognosis of the high infiltration group of resting CD4 memory T cells was better than that of the low infiltration group (*P* = .002, Log-rank statistic = 4.139). The prognosis of the macrophage M0 high infiltration group was worse than that of the low infiltration group (*P* = .026, Log-rank statistic = 3.553). The prognosis of the activated mast cell high infiltration group was worse than that of the low infiltration group (*P* = .026, Log-rank statistic = 3.740). As shown in Figure 3.

### 3.4. Single factor COX analysis of immune cell content and prognosis

Univariate COX analysis showed that dormant dendritic cells, eosinophils, resting mast cells, monocytes, and resting CD4 memory T cells were the protective factors for the prognosis of breast cancer patients (hazard ratio [HR] < 1, *P* < .05). Macrophage M0 and activation of mast cells are risk factors for the prognosis of breast cancer patients (HR > 1, *P* < .05). As shown in Table 1.

**Table 1:** Single factor Cox analysis of immune cell infiltration and prognosis.

Immune cell	HR	95%CI		*P* value
		Upper limit	Lower limit	
B cells naive	1.34	0.86	1.94	.231
B cells memory	0.83	0.52	1.03	.920
Plasma cells	1.22	0.73	1.51	.698
T cells CD8	1.78	0.46	2.29	.346
T cells CD4 naive	1.45	0.36	2.11	.864
T cells CD4 memory resting	0.66	0.34	1.27	.008
T cells CD4 memory activated	0.48	0.14	1.07	.135
T cells follicular helper	0.64	0.45	0.93	.474
T cells regulatory (Tregs)	1.29	0.90	1.65	.358
T cells gamma delta	1.32	0.95	2.29	.663
NK cells resting	1.84	0.79	2.01	.961
NK cells activated	1.25	0.57	1.64	.523
Monocytes	0.68	0.14	0.73	.034
Macrophages M0	1.34	0.86	1.87	.016
Macrophages M1	0.42	0.34	1.32	.844
Macrophages M2	0.74	0.53	0.65	.051
Dendritic cells resting	0.63	0.22	1.17	.036
Dendritic cells activated	1.35	0.76	2.01	.913
Mast cells resting	0.41	0.20	1.16	.014
Mast cells activated	1.86	1.23	2.75	.005
Eosinophils	0.59	0.21	0.89	.015
Neutrophils	1.21	0.64	2.18	.481

CI = means confidence interval, HR = refers to hazard ratio.

### 3.5. Multivariate COX analysis of immune cell content and prognosis

Multivariate COX analysis showed that resting mast cells were independent prognostic factors for breast cancer (HR < 1, *P* < .05). Macrophage M0 and activation of mast cells are independent risk factors for the prognosis of breast cancer (HR > 1, *P* < .05). As shown in Table 2.

**Table 2:** Multivariate COX analysis screening for independent prognostic factors.

Immune cell	HR	95%CI		*P* value
		Upper limit	Lower limit	
B cells naive	1.03	0.72	1.53	.373
B cells memory	0.67	0.44	1.21	.864
Plasma cells	1.21	0.86	1.45	.548
T cells CD8	1.62	0.63	1.84	.671
T cells CD4 naive	1.31	0.59	1.65	.333
T cells CD4 memory resting	0.71	0.48	1.42	.417
T cells CD4 memory activated	0.88	0.59	1.23	.245
T cells follicular helper	0.73	0.65	1.41	.368
T cells regulatory (Tregs)	1.09	0.72	1.50	.401
T cells gamma delta	1.14	0.83	2.01	.634
NK cells resting	1.53	0.91	1.84	.377
NK cells activated	1.18	0.62	1.51	.469
Monocytes	0.71	0.58	1.11	.124
Macrophages M0	1.20	0.77	1.49	.039
Macrophages M1	0.81	0.50	1.29	.146
Macrophages M2	0.77	0.61	0.96	.136
Dendritic cells resting	0.71	0.22	0.91	.148
Dendritic cells activated	1.21	0.71	1.59	.216
Mast cells resting	0.64	0.49	0.93	.022
Mast cells activated	1.55	1.04	2.18	.010
Eosinophils	0.83	0.72	1.63	.324
Neutrophils	1.06	0.79	1.54	.089

CI = means confidence interval, HR = refers to hazard ratio.

### 3.6. Screening of genes related to prognostic immune cells

**Figure 4.**
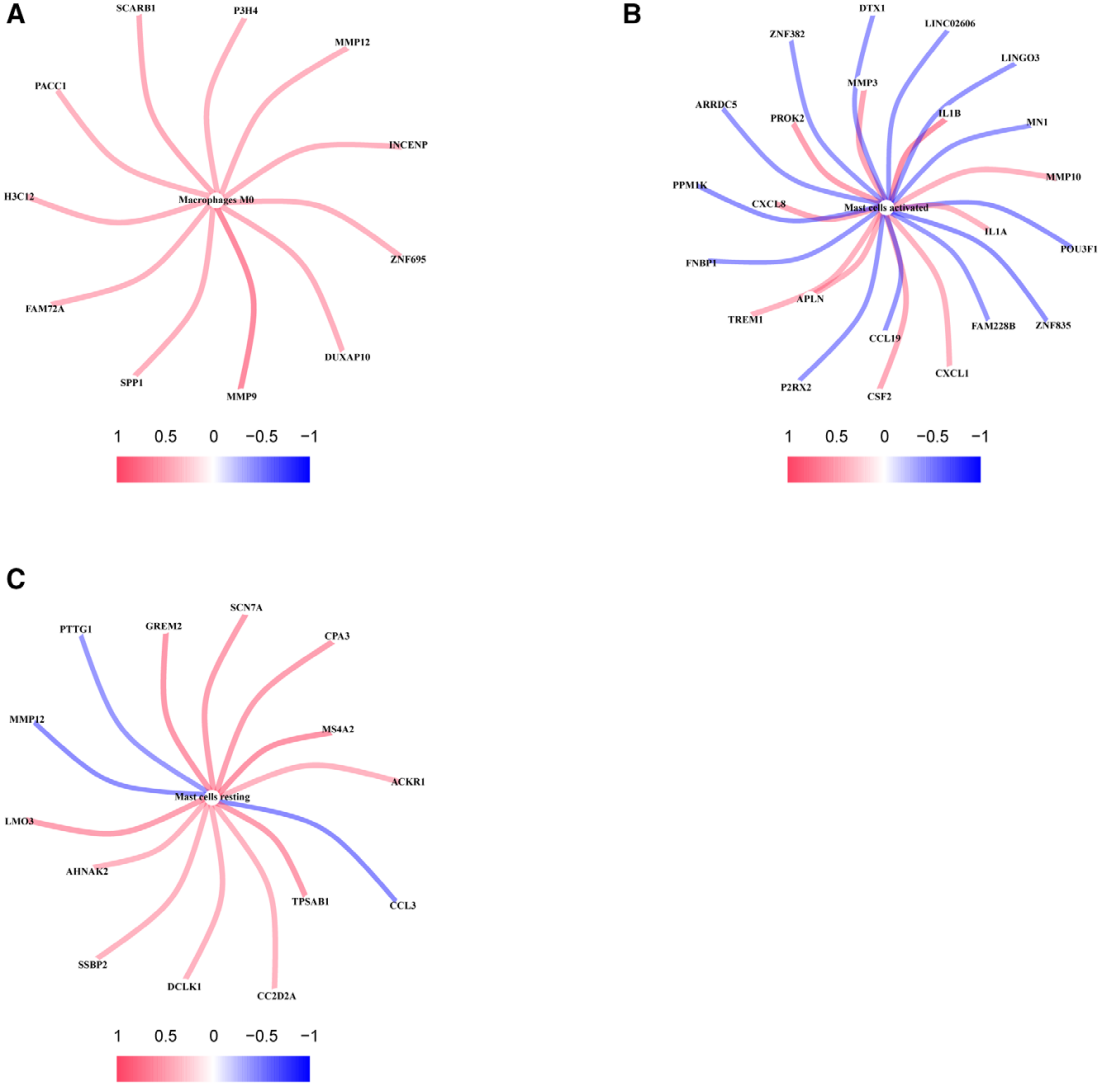
Genes related to prognostic immune cells. (A) Macrophage M0; (B) mast cells activated; (C) mast cells resting.

We used correlation coefficient > 0.4 and *P* < .001 as screening criteria to screen out genes related to prognostic immune cells, as shown in Figure 4A–C.

### 3.7. Epithelial-mesenchymal transition (EMT) and cancer stem cells

**Figure 5.**
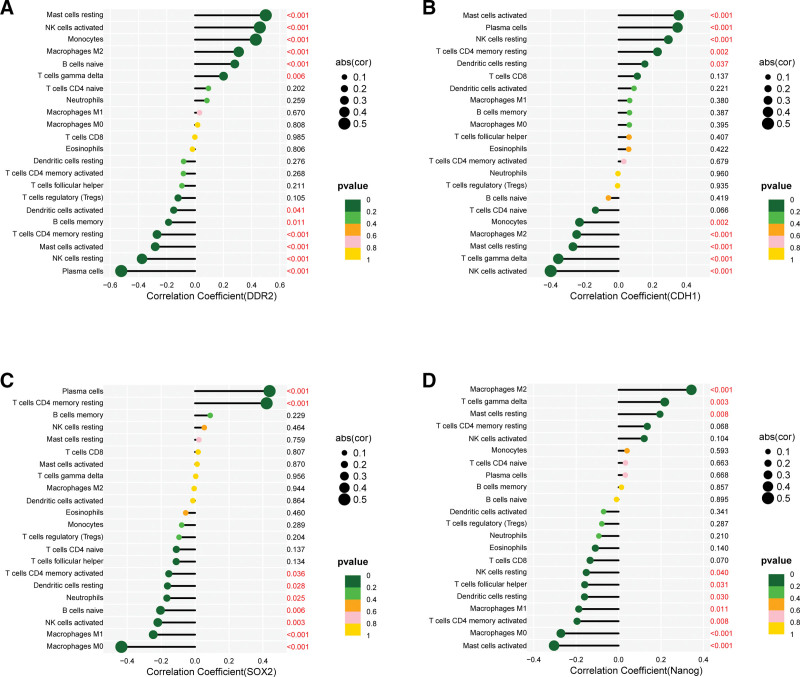
The relationship between immune cells and markers of epithelial-mesenchymal transition and markers of breast cancer stem cells.

DDR2 and CDH1 are the markers of EMT. DDR2 was positively correlated with mast cells resting, and negatively correlated with mast cells activated (Fig. 5A). CDH1 was positively correlated with mast cells resting, and negatively correlated with mast cells activated (Fig. 5B). SOX2 and Nanog are the markers of cancer stem cells. DDR2 was negatively correlated with macrophage M0 (Fig. 5C). Nanog was positively correlated with mast cells resting, and negatively correlated with mast cells activated and macrophage M0 (Fig. 5D).

## 4. Discussion

Tumor tissue comprises tumor cells, immune cells, fibroblasts and cytokines. These elements interact to form the tumor microenvironment, which is closely related to the occurrence, development, and metastasis. ICI has been proved to be associated with the biological behavior of cancer cells and the clinical characteristics of tumors. Previous studies have used immunohistochemistry and flow cytometry to explore the role of ICI in tumor treatment, but these strategies have their limitations. Immunohistochemistry relies on cellular protein markers to identify immune cell subsets. This technique can only identify a small part of these subsets, and the results may be biased because the markers also exist in other types of cells. Flow cytometry relies on various protein markers to identify immune cell subtypes. However, the results are limited by the fluorescence channel. Therefore, most previous studies could not determine the effects of multiple specific immune cell subtypes on tumor occurrence, progression, prognosis and treatment. CIBERSORT provides an excellent solution to overcome these limitations. CIBERSORT is a complex deconvolution algorithm based on gene expression data, famous for its high resolution.^[15]^ The algorithm uses 500 marker genes, including specific immune cell markers, for prognostic evaluation and treatment strategy selection. The algorithm has been verified by fluorescence-activated cell classification and immunohistochemical staining. Studies have confirmed through the CIBERSORT algorithm that neoadjuvant chemotherapy can change the tumor microenvironment, immune cells are related to the prognosis of tumor patients, and the infiltration abundance of immune cells determines the sensitivity of tumor patients to chemotherapy and radiotherapy.^[16,17]^

Our study found that follicular T cells, regulatory T cells, and macrophages M0 infiltration in breast cancer tissues were higher than those in normal breast tissues. The contents of activated mast cells and eosinophils were lower than those in normal tissues. We also identified independent risk factors for breast cancer prognosis, including macrophages M0 and activated mast cells, which play a key role in the occurrence and progression of breast cancer. Our research is consistent with some other research results. Studies have shown that the distribution of macrophage M0 in breast cancer tissues is diverse and heterogeneous. The distribution of macrophage M0 in breast cancer tissues is positively correlated with VEGF and MVD, which promotes breast cancer progression by regulating angiogenesis in breast cancer.^[18]^ Studies also have shown that macrophage M0 may reduce the sensitivity of breast cancer cells to chemotherapeutic drugs by producing interleukin 10.^[19]^ A previous study treated breast cancer cells with a macrophage M0 culture medium supernatant. Researchers observed enhanced biological behaviors, mainly the proliferation, invasion and migration ability, in breast cancer cells treated with macrophage M0 medium.^[20]^ Few studies illustrated the effect of activated mast cells on the biological behavior of cancer cells in the past. Some studies believe mast cells can secrete histamine and promote tumor invasion and migration.^[21–23]^ Our study found that resting mast cells are independent prognostic factors for breast cancer patients. Activated mast cells and dormant mast cells are negatively correlated. The results of correlation analysis also confirmed this view, and the correlation coefficient of the 2 groups was r = −0.36. Therefore, resting mast cells may play a protective role in breast cancer prognosis by reducing histamine secretion. Survival analysis showed that high macrophage M2 predicted a good prognosis. This may be an anomaly. Macrophage M2 inhibits the inflammatory response. This result was not demonstrated in univariate and multivariate COX analyses. We cannot make a hasty conclusion about the correlation between macrophage M2 and prognosis. We found that prognostic immune cells showed a correlation with EMT markers and tumor stem cell markers. This was a phenomenon worth exploring in depth.

There were still some flaws in this study. First, due to data limitations, this study failed to clarify the relationship between ER/PR/HER2 status and immune cells. Second, this study lacks validation from external data. Finally, this study was not validated in vivo or in vitro. We will carry out continuous work to address these deficiencies.

### Conclusion:

In this study, we have made a macroscopic description of the infiltration of immune cells in breast cancer. We have identified macrophages M0 and activated mast cells, which may be related to the occurrence and progression of breast cancer and provide clues for finding the targets of immunotherapy and the interaction mechanism between immune cells and tumor cells.

## Author contributions

**Conceptualization:** Yuan-Yuan Zhang.

**Data curation:** Yi-Min Gan.

**Formal analysis:** Yuan-Yuan Zhang, Yi-Min Gan.

**Funding acquisition:** Yuan-Yuan Zhang, Yi-Min Gan.

**Investigation:** Yuan-Yuan Zhang, Yi-Min Gan.

**Methodology:** Yuan-Yuan Zhang, Yi-Min Gan.

**Project administration:** Yuan-Yuan Zhang, Yi-Min Gan.

**Resources:** Yuan-Yuan Zhang, Yi-Min Gan.

**Software:** Yuan-Yuan Zhang, Yi-Min Gan.

**Supervision:** Yuan-Yuan Zhang, Yi-Min Gan.

**Validation:** Yuan-Yuan Zhang, Yi-Min Gan.

**Visualization:** Yuan-Yuan Zhang, Yi-Min Gan.

**Writing – original draft:** Yuan-Yuan Zhang, Yi-Min Gan.

**Writing – review & editing:** Yuan-Yuan Zhang, Yi-Min Gan.
